# Gelling Characteristics and Mechanisms of Heat-Triggered Soy Protein Isolated Gels Incorporating Curdlan with Different Helical Conformations

**DOI:** 10.3390/foods14142484

**Published:** 2025-07-16

**Authors:** Pei-Wen Long, Shi-Yong Liu, Yi-Xin Lin, Lin-Feng Mo, Yu Wu, Long-Qing Li, Le-Yi Pan, Ming-Yu Jin, Jing-Kun Yan

**Affiliations:** 1 Engineering Research Center of Health Food Design & Nutrition Regulation, Dongguan Key Laboratory of Typical Food Precision Design, China National Light Industry Key Laboratory of Healthy Food Development and Nutrition Regulation, School of Life and Health Technology, Dongguan University of Technology, Dongguan 523808, China; 2 College of Food Science, South China Agricultural University, Guangzhou 510642, China

**Keywords:** soy protein isolate, curdlan, gelling property, helical conformations, functional properties

## Abstract

This study investigated the effects of curdlan (CUR) with different helical conformations on the gelling behavior and mechanisms of heat-induced soy protein isolate (SPI) gels. The results demonstrated that CUR significantly improved the functional properties of SPI gels, including water-holding capacity (0.31–5.06% increase), gel strength (7.01–240.51% enhancement), textural properties, viscoelasticity, and thermal stability. The incorporation of CUR facilitated the unfolding and cross-linking of SPI molecules, leading to enhanced network formation. Notably, SPI composite gels containing CUR with an ordered triple-helix bundled structure exhibited superior gelling performance compared to other helical conformations, characterized by a more compact and uniform microstructure. This improvement was attributed to stronger hydrogen bonding interactions between the triple-helix CUR and SPI molecules. Furthermore, the entanglement of triple-helix CUR with SPI promoted the formation of a denser and more homogeneous interpenetrating polymer network. These findings indicate that triple-helix CUR is highly effective in optimizing the gelling characteristics of heat-induced SPI gels. This study provides new insights into the structure–function relationship of CUR in SPI-based gel systems, offering potential strategies for designing high-performance protein–polysaccharide composite gels. The findings establish a theoretical foundation for applications in the food industry.

## 1. Introduction

Soybean protein isolate (SPI) is a plant-derived protein that comprises more than 90% of the total protein content and is obtained from soybean meal, excluding oil and water-soluble non-protein constituents [1]. SPI offers various advantages, including a rich nutritional profile, balanced amino acid composition, affordability, and ready availability [2]. It serves as a base ingredient for the production of diverse protein-based food products, including soybean protein beverages, baked goods, and meat substitutes for vegetarians [3,4]. Furthermore, its sustainable sourcing has led to its gradual substitution for animal-based proteins, particularly in the creation of plant-based meat alternatives [5,6]. The functionality of SPI-based food products hinges on their exceptional attributes, including solubility, emulsifiability, foamability, water-holding capacity (WHC), oil-holding capacity, and, most notably, gel properties [7]. However, pure SPI gels with multiple subunits (7S/β-associated soy globulin, 11S/glycinin, etc.) exhibit limited elasticity and a loose structure, making them unsuitable for the demands of food processing [8]. Additionally, protein gels are susceptible to fluctuations in external conditions, such as salt concentration, pH, and temperature, which considerably restrict their practical utility within the food industry [9]. Consequently, enhancing the gelling characteristics of SPI gels holds significant importance for the development of innovative food products.

In essence, the gel properties of SPI gels arise from a process involving protein unfolding, denaturation, and subsequent aggregation, culminating in the formation of cross-linked networks [6]. Among the available methods for improving SPI gel properties, heat treatment and chemical modification are mild and convenient approaches [10]. Heat-induced SPI undergoes cross-linking of molecular chains through hydrogen bonds and van der Waals forces, resulting in the formation of uniform and compact gel networks with remarkable stability. Numerous studies have demonstrated that the gelling properties of heat-induced SPI gels can be further enhanced through the incorporation of exogenous food polysaccharides. Examples of these include oat β-glucan [11], sugar beet pectin (SBP) [12], wheat bran cellulose (WBC) [13], soybean polysaccharide (SSPS) [14], and *Lycium barbarum* polysaccharide (LBP) [15], which interact with SPI, contributing to the gel-forming functional properties and nutritional benefits. Notably, curdlan (CUR), a bacterial exopolysaccharide derived from the fermentation of *Alcaligenes faecalis* that is composed of linear β-1,3-glucan, has gained recognition as a valuable food additive [16,17]. Following FDA approval, CUR has been extensively applied in the food industry, primarily owing to its outstanding gel-forming properties [18]. Recent research by Li et al. revealed that the incorporation of thermo-reversible CUR (TRC) or thermo-irreversible CUR (TIRC) gels can evidently enhance the gelling properties of heat-induced SPI gels [19]. Notably, TRC, characterized by a stable triple-helix structure, exhibits superior improvements in thermal gelling and structural properties compared to TIRC, which features a single-helix structure. It is crucial to note that the type, structural characteristics, and chain conformations of polysaccharides play pivotal roles in enhancing the gelling properties and elucidating the gelation mechanism of heat-induced SPI gels. Furthermore, research by Xiao et al. underscores the influence of heating temperatures on the helical conformations of CUR molecules [20]. The bundled triple-stranded helix chains of CUR were driven to separate from each other from 25 °C to 50 °C, and partially opened triple-helical chains from 60 °C to 70 °C, finally into a dissociated single-helix chain from 80 °C to 90 °C. In light of these findings, it is hypothesized that the addition of CUR with varying helical conformations will exert notable effects on the gelling properties and gelation mechanism of heat-induced SPI gels.

Hence, the primary objective of this study was to investigate the influence of the inclusion of CUR on the physiochemical and gelling properties of SPI gels subjected to thermal treatment. The effects of CUR with different helical conformations on the gel strength, WHC, textural characteristics, rheological properties, microstructures, and thermal stability of CUR-SPI composite gels were analyzed and compared. In addition, the potential gelation mechanisms of CUR-SPI composite gels were elucidated. The present study will offer a potential avenue for enhancing the gelling characteristics of heat-induced SPI gels.

## 2. Materials and Methods

### 2.1. Materials and Chemicals

SPI (protein content: 94.5%) was obtained from Shanghai Yuanye Biotechnology Co., Ltd. (Shanghai, China). CUR was purchased from Wako Pure Chemical Co. (Tokyo, Japan). NaOH and HCl were analytically pure and bought from Sinopharm Chemical Reagent Co., Ltd. (Shanghai, China).

### 2.2. Preparation of CUR Solutions with Different Helical Conformations

In accordance with the procedure outlined by Xiao et al. [20], 0.2 g of CUR powder was dispersed in 20 mL of distilled water and subjected to magnetic stirring at a temperature of 25 °C for 12 h. Subsequently, the resulting CUR suspension was incubated at four distinct temperatures, 40 °C, 50 °C, 60 °C, and 80 °C, within a water bath for a period of 2 h, while continuous stirring was maintained. This process yielded CUR solutions (1.0%, *w*/*v*) characterized by varying helical conformations, which were subsequently utilized in the preparation of CUR-SPI mixed gels.

### 2.3. Preparation of CUR-SPI Composite Gels

The preparation of the CUR-SPI mixed gels was performed in accordance with Xiao et al.’s methodology [13], albeit with minor adjustments. To summarize, SPI powder was dissolved in distilled water and subjected to magnetic stirring at 25 °C for 2 h by a magnetic stirrer (Heidolph Instruments GmbH & Co. KG, Schwabach, Germany), followed by overnight refrigeration at 4 °C to ensure complete hydration. This process yielded an 18% (*w*/*v*) SPI solution. Subsequently, the SPI solution was heated to 80 °C for 30 min under continuous stirring, after which it was individually cooled to 60 °C, 50 °C, and 40 °C by a low-temperature cooling circulation system (DKL-5003, Ningbo Scientz Biotechnology Co., Ltd., Ningbo, China), resulting in four distinct SPI solutions designated SPI_80_, SPI_60_, SPI_50_, and SPI_40_, respectively. Next, 20 mL of pre-prepared 1% (*w*/*v*) CUR solution, corresponding to each of the respective temperatures (80 °C, 60 °C, 50 °C, and 40 °C), was gently introduced into 20 mL of the prepared SPI solution. The pH of the mixture was adjusted to 7.0 by using NaOH and HCl, followed by magnetic stirring for 30 min under each of the respective temperatures (80 °C, 60 °C, 50 °C, and 40 °C). Subsequently, the mixture was cooled to room temperature and left to incubate at 4 °C overnight for further analysis. The resulting CUR-SPI composite gels obtained at different temperatures (40 °C, 50 °C, 60 °C, and 80 °C), each with a final SPI concentration of 9%, were denoted as CUR-SPI_40_, CUR-SPI_50_, CUR-SPI_60_, and CUR-SPI_80_, respectively. As a reference, a heat-induced SPI gel with a final concentration of 9% was prepared at 80 °C without CUR and served as a blank control.

### 2.4. WHC

The gel sample was accurately weighed and transferred to a centrifuge tube, which was then subjected to centrifugation at 10,000× *g* and 4 °C for 15 min by a centrifuge (Hunan Cenlee Scientific Instruments Co., Ltd., Changsha, China). After centrifugation, any residual water was thoroughly removed using filter paper, and the weight of the gel was recorded. The WHC (%) is expressed as a percentage and was defined as the ratio of the gel’s mass after centrifugation (*m*_2_) to its initial mass (*m*_1_) and can be computed using the following formula (Equation (1)) [21]:

WHC (%) = (m_2_/m_1_) × 100% (1)

### 2.5. Gel Strength

Gel strength was assessed using a TA-XT Plus texture analyzer (Stable Micro Systems Ltd., Godalming, UK) as the previously described method in [13]. Prior to measurement, gel samples were equilibrated at room temperature for 30 min and molded into cylindrical specimens (2.5 cm diameter × 2.5 cm height). Each specimen was then penetrated using a P/0.5 spherical probe at a constant speed of 1 mm/s to a depth of 10 mm. The test was triggered by a 0.05 N force, with a pre-test and post-test speed of 1 mm/s. A 50 N load cell was used, and the probe returned to a distance of 35 mm after each test. Gel strength is defined as the peak force (N) at 10 mm penetration depth.

### 2.6. Texture Profile Analysis (TPA)

Following the procedure described by Li et al. [14], gel hardness, chewiness, springiness, and cohesiveness were assessed using a TA-XT Plus texture analyzer (Stable Micro Systems Ltd., Godalming, UK) equipped with a P36R spherical probe. Cylindrical gel samples (36 mm diameter × 10 mm height) were tested at 25 °C. A 5 N load cell was used for the uniaxial compression test, in which samples were compressed to 30% deformation at a rate of 1 mm/s. The stress-strain relationship was recorded during compression.

### 2.7. Dynamic Rheological Measurements

The dynamic rheological properties of both the SPI and CUR-SPI gels were assessed using a rheometer (MCR702, Anton Paar, Graz, Austria) equipped with a parallel plate (diameter: 40 mm, gap: 1 mm) following a previously established methodology with minor adjustments [22]. The SPI and CUR-SPI suspensions were introduced onto the test platform and encircled with silicone oil to prevent evaporation throughout the experiment. After equilibration at 25 °C for 15 min, oscillating temperature sweeps were conducted. These sweeps included temperatures ranging from 25 °C to 80 °C and subsequently from 80 °C to 25 °C. The heating and cooling were carried out at a consistent rate of 2 °C/min. An amplitude sweep test of gel samples was performed at 80 °C with a strain range from 0.1 to 100% at a constant angular frequency of 1 Hz to confirm the linear viscoelastic region. A constant angular frequency of 1 Hz and a fixed strain of 1% were used to record the storage modulus (G′) and loss modulus (G″).

### 2.8. Scanning Electron Microscopy (SEM) Observation

SEM was performed slightly modified in reference to Xiao et al. [13]. The gel sample was promptly frozen using liquid nitrogen and subsequently subjected to freeze-drying at −50 °C for 48 h by a freeze dryer (Ningbo Scientz Biotechnology Co., Ltd., Ningbo, China). After the lyophilization process, the freeze-dried gel sample was coated with a layer of gold, and its microstructure was examined using a scanning electron microscope (SEM, EM-30 plus, COXEM Co., Ltd., Daejeon, Republic of Korea). SEM analysis was conducted at an accelerating voltage of 20 kV, with the magnification set to 200×.

### 2.9. Thermogravimetric Analysis (TGA)

According to the reported method [23], the thermal properties of the SPI and CUR-SPI gels were evaluated via a thermogravimetric analyzer (TGA 8000, Perkin Elmer Instruments Co., Ltd., Waltham, MA, USA). The freeze-dried gel sample (5.0 mg) was heated from 30 °C to 600 °C at 10 °C/min under a 20 mL/min flow of dry nitrogen.

### 2.10. Fourier Transform Infrared (FT-IR) Spectroscopy

The examined sample was combined with KBr powder at a mass ratio of 1:100 (*w*/*w*) and subsequently compressed into a transparent sheet with a thickness of 1 mm. The FT-IR spectrum was acquired using an FT-IR spectrometer (Cary 5000, Agilent Technologies, Santa Clara, CA, USA), covering the wavenumber range from 400 to 4000 cm^−1^ at a resolution of 4 cm^−1^ [13].

### 2.11. Circular Dichroism (CD) Spectroscopy

Following the reported method [19], the secondary structure of the SPI was examined utilizing a circular dichroism (CD) spectrometer (Chirascan V100, Applied Photophysics Ltd., Surrey, UK). To outline the procedure briefly, lyophilized SPI and CUR-SPI gels were dissolved in distilled water, stirred using magnetic agitation at room temperature for 2 h, and subsequently subjected to centrifugation at 10,000× *g* for 15 min. The resulting supernatant, with a concentration of 0.5 mg/mL SPI, was then subjected to scanning within the wavelength range of 180–260 nm, specifically in the far ultraviolet region.

### 2.12. Fluorescence Spectroscopy

A fluorescence spectrophotometer (F-7000, Hitachi, Tokyo, Japan) was used to analyze the intrinsic fluorescence of the gel sample in accordance with a previous study [24]. To provide a succinct overview, solutions of SPI and CUR-SPI, each with a concentration of 0.5 mg/mL SPI, were prepared as described in Section 2.11. The excitation wavelength utilized was 280 nm, while the emission wavelength was scanned over the range of 300–500 nm. Scanning was conducted at a speed of 300 nm/min, and both the excitation and emission slits were set at 5.0 nm.

### 2.13. Statistical Analysis

This study is a single-factor experimental design. All the data were subjected to a minimum of three independent measurements, and the results are presented as the mean ± standard deviation (SD). To determine significant differences (*p* < 0.05), one-way analysis of variance (ANOVA) was conducted, followed by Turkey’s test for post hoc analysis. Data analysis and graphical representation were performed by using Origin 2021 software (OriginLab Corp., Northampton, MA, USA).

## 3. Results and Discussion

### 3.1. WHC and Gel Strength

Jiang et al. [20] systematically investigated the thermally induced conformational transitions of curdlan, revealing its temperature-dependent structural evolution. The results demonstrated that CUR undergoes a series of characteristic conformational changes upon heating: hydration and swelling dominate at 40 °C, the bundled triple-helix strands begin to dissociate at 50 °C, partially opened triple helices and single helices form between 60 and 70 °C, and dissociation into single helical chains occurs above 80 °C. Based on the above findings, we further explored the influence of different CUR conformations on the thermally induced gelation behavior of soy protein isolate.

**Figure 1:**
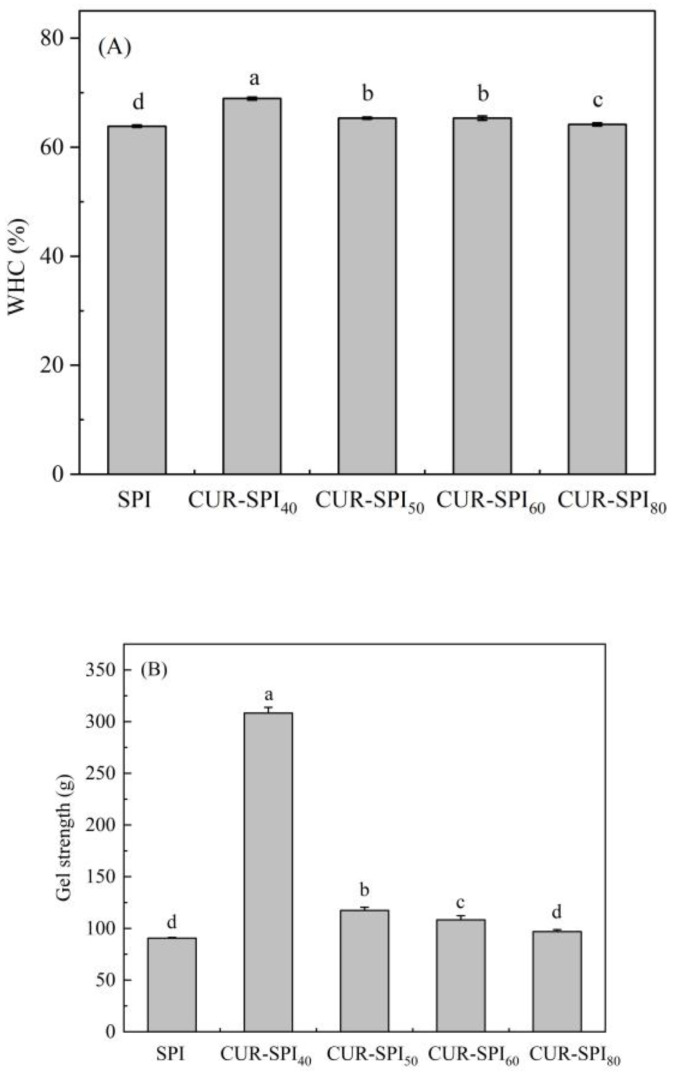
(**A**) WHC and (**B**) gel strength of SPI and CUR-SPI gels. Each value was represented as mean ± SD (*n* = 3), and different lowercase letters signified significant differences (*p* < 0.05).

The WHC serves as a crucial parameter for assessing the quality of a gel system. For rigid gel systems, a higher WHC signifies greater water retention within the gel system, consequently enhancing gel elasticity [25]. Additionally, the WHC is largely influenced by the microstructure and gel strength [26]. Figure 1A depicts the effects of CUR with various helical conformations on the WHC of heat-induced SPI gels. As anticipated, the incorporation of CUR obviously enhanced the WHC of the SPI gels compared to that of the pure SPI gel (*p* < 0.05). The specific performance shows that CUR-SPI_40_ increased by 5.06%, CUR-SPI_50_ rose by 1.45%, CUR-SPI_60_ improved by 1.45%, and CUR-SPI_80_ grew by 0.31%. This increase in the WHC of the heat-induced SPI gels can be attributed to the formation of intermolecular hydrogen bonds between the hydroxyl groups in the hydrophilic CUR and water molecules [13,25]. Additionally, this process results from the establishment of a well-structured three-dimensional gel network, allowing for the physical inclusion of a greater number of water molecules [13]. These findings align with the findings of Xing et al. [15], who highlighted the role of hydrophilicity in the water retention of composite SPI-LBP gels, consistent with the results of Chen et al. [12] and our present investigation. Notably, Figure 1B reveals that among the CUR-SPI mixed gels, CUR-SPI_40_ exhibited the highest WHC, 68.93%. This observation suggests that CUR-SPI_40_ mixed gels prepared by adding CUR with triple-helix structural bundles retained more water molecules and thus increased the WHC as compared to the addition of CUR with other conformations. This is attributed to its superior swelling and hydration capability [20]. A similar effect was reported by Jiang et al. [27], where the addition of TRC with a triple-helix structure resulted in a greater WHC for myofibrillar protein (MP) composite gels than did the addition of TIRC with a single-helix structure at the same concentration.

Gel strength, another vital parameter of the gel system, reflects the aggregation ability of the protein gel during thermal denaturation. Figure 1B illustrates the effect of the addition of CUR with various helical conformations on the gel strength of heat-induced SPI gels. It is evident that the gel strength of the CUR-SPI mixed gels was significantly greater than that of the single SPI gel (*p* < 0.05). The gel strength of CUR-SPI40 increased by 240.51%, CUR-SPI50 rose by 29.66%, CUR-SPI60 improved by 19.55%, and CUR-SPI80 grew by 7.01%. The increase in gel strength is typically attributed to the ordered arrangement of protein networks [13]. In the present study, the increased gel strength of the SPI gels may be attributed to changes in protein-protein interactions and protein aggregation induced by the addition of CUR [28]. In particular, as depicted in Figure 1B, the CUR-SPI_40_ mixed gels prepared by the addition of CUR with triple-helix bundles have the highest gel strength (3.08 N) among the CUR-SPI composite gels. With increasing temperature, CUR underwent a transition from hydration and swelling of bundled triple helixes (40 °C) to the separation of triple helixes (50 °C), partial dissociation (60 °C), and ultimately a single-helix state (80 °C) [20]. This corresponded to an overall increase in disorderliness in the assembly state of CUR in solution [20]. Jiang et al.’s work also supports these findings [27], demonstrating that TRC with an ordered triple-helix structure led to greater gel strength in MP mixed gels than TIRC did with a disordered single-helix chain, aligning with our current study and the results reported by Li et al. [19]. In summary, the results from Figure 1 collectively indicate that the addition of CUR with an ordered triple-helix structure in bundles noticeably enhances the WHC and gel strength of heat-induced SPI gels.

### 3.2. TPA Characteristics

Texture profile analysis (TPA) is a common method for assessing the mechanical characteristics of food gels [29]. Table 1 shows the effects of incorporating CUR with different helical conformations on the textural properties, including hardness, chewiness, springiness, and cohesiveness, of heat-induced SPI gels. Overall, in comparison to the pure SPI gel, the introduction of CUR significantly enhanced the hardness, chewiness, and springiness of the SPI gels (*p* < 0.05), with the exception of CUR-SPI_80_, which had no significant influence on cohesiveness (*p* > 0.05). The enhancement in the textural properties of the SPI gels can be attributed to several factors. First, the addition of CUR effectively immobilized a substantial quantity of water molecules, thereby increasing its binding to SPI and improving SPI concentration. This was conducive to the formation of viscoelastic gels with improved mechanical properties. Additionally, the neutral CUR, which is rich in hydroxyl groups, could establish physically cross-linked interpenetrating gel networks with the amino groups of SPI through hydrogen bonding [25]. This interaction contributed to the enhancement of the mechanical properties of the CUR-SPI composite gels. Comparable results have been observed for double network gels based on SPI and SBP [12], as well as for SPI xerogels modulated by SSPS [14]. Moreover, as detailed in Table 1, CUR-SPI_40_ exhibited the highest hardness (3.20 N), chewiness (7.10 N), and springiness (97.52%). In contrast, CUR-SPI_80_ displayed the lowest values among the CUR-SPI composite gels. This result suggested that CUR obtained at 40 °C, characterized by bundles of triple helices, underwent tighter physical cross-linking with thermally denatured SPI, resulting in a denser complex gel network. These findings align with the observations regarding WHC (Figure 1A) and gel strength (Figure 1B). Conversely, CUR obtained at 80 °C was dominated by a disordered single helical conformation, primarily acting as a filler within the SPI gel network [27], and its impact on textural properties was not significant (*p* > 0.05, Table 1).

### 3.3. Dynamic Rheological Properties

**Figure 2:**
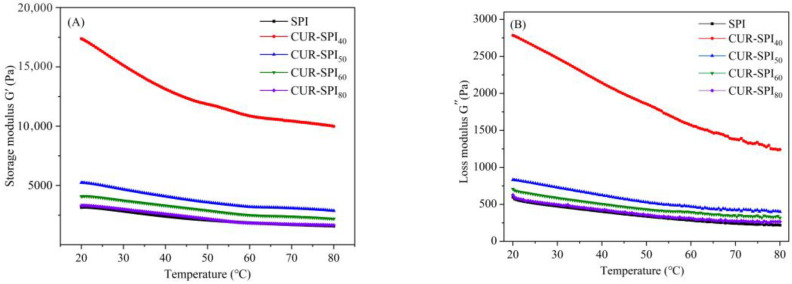

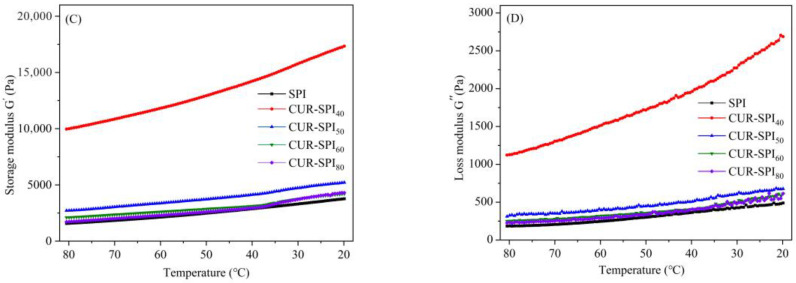
Dynamic rheological properties of pure SPI and CUR-SPI gels. (**A**) Storage modulus *G*′ and (**B**) loss modulus *G*″ during the heating process. (**C**) Storage modulus *G*′ and (**D**) loss modulus *G*″ during the cooling process.

The process of forming CUR-SPI composite gels during heating in a water bath at 80 °C was simulated by analyzing the changes in the storage modulus (*G*′) and loss modulus (*G*″) during heat treatment. As depicted in Figure 2, the incorporation of CUR elevated both the *G*′ and *G*″ values in the SPI gels compared to those in the pure SPI gel during the heating and cooling processes, presenting a soft solid-type viscoelastic behavior. This effect was particularly pronounced when CUR was used at 40 °C, which is characterized by bundles of triple helices. This finding suggests that, relative to other CUR-SPI composite gels, the CUR-SPI_40_ mixed gels prepared by the addition of CUR with triple-helix bundles resulted in the formation of superior three-dimensional network structures. Furthermore, the *G*′ values of both the SPI and CUR-SPI gels were considerably greater than the *G*″ values across the measured temperature range (25–80 °C). This indicates that the addition of CUR enhances the dynamic viscoelastic properties of SPI gels, predominantly resulting in solid elasticity. These results align with the findings of Li et al. [19], who demonstrated that the addition of TRC led to higher *G*′ values in the SPI gel system than the addition of TIRC did. Similar observations have been reported by Jiang et al. [27]. In summary, these results collectively highlight that CUR, particularly CUR obtained at 40 °C, which features bundles of triple-helix structures, can serve as a rheological enhancer to improve the viscoelasticity of SPI gels. This observation substantiates the enhanced gel strength and WHC observed in the CUR-SPI composite gels.

### 3.4. Microstructures

**Figure 3:**
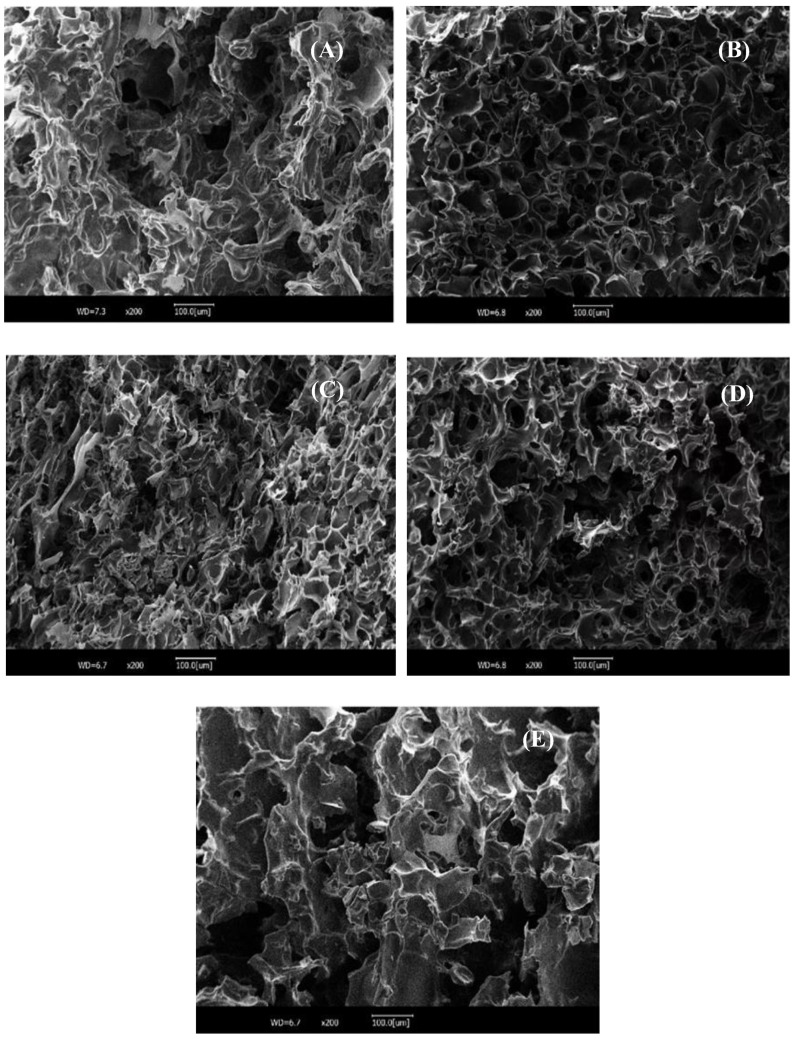
SEM images of (**A**) SPI, (**B**) CUR-SPI_40_, (**C**) CUR-SPI_50_, (**D**) CUR-SPI_60_, and (**E**) CUR-SPI_80_ gels at a magnification of 200×. The CUR-SPI_40_, CUR-SPI_50_, CUR-SPI_60_, and CUR-SPI_80_ were the CUR-SPI composite gels obtained at different temperatures (40 °C, 50 °C, 60 °C, and 80 °C).

SEM was employed in this study to examine the microstructures of the SPI gels with and without CUR addition, as depicted in Figure 3. As shown in Figure 3A, the pure SPI gel displayed coarse surfaces and broken and disordered structures, as did the loose and sizable cavities. This observation may be attributed to the inadequate unfolding of SPI molecules and their insufficient bonding with neighboring SPI molecules during thermal gelation [13]. As anticipated, compared to that of the SPI gel alone (Figure 3A), the incorporation of CUR in various helical conformations (Figure 3B–E) resulted in relatively homogeneous, ordered, and well-structured three-dimensional networks with compact and small pores in the CUR-SPI mixed gels. These observations strongly support the enhanced mechanical properties (Table 1) and improved WHC (Figure 1A) of the CUR-SPI mixed gels, further enhancing the quality of the gel system. These findings are consistent with those of a report by Xiao et al. [13], who demonstrated that the addition of a hydrophilic WBC led to the formation of more regular and uniform network structures with smooth surfaces and denser cavities in heat-induced SPI gels. Furthermore, it is worth noting that among the CUR-SPI composite gels, CUR-SPI_40_ (Figure 3B) exhibited the formation of even more uniform and regular network structures with more compact and orderly pores. Combining the results of gel strength and the WHC, CUR-SPI_40_ have the highest hardness and cohesiveness. These findings align with the results reported by Li et al. [14], Jiang et al. [27], and Wu et al. [30].

### 3.5. Thermal Stability

**Figure 4:**
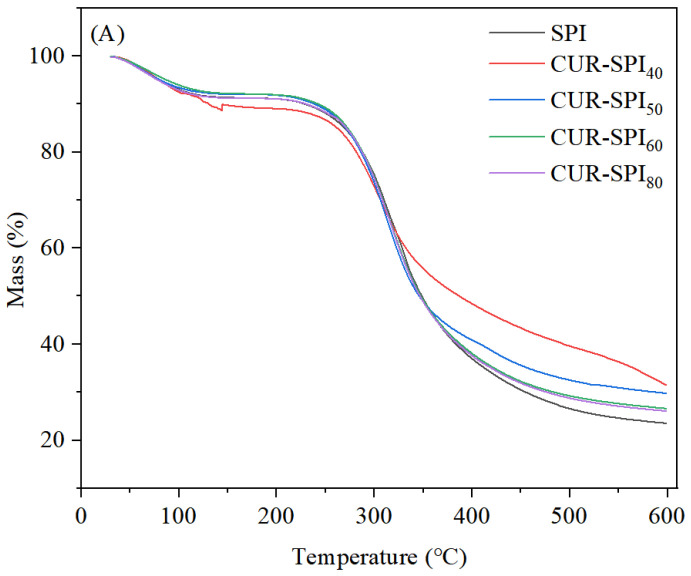

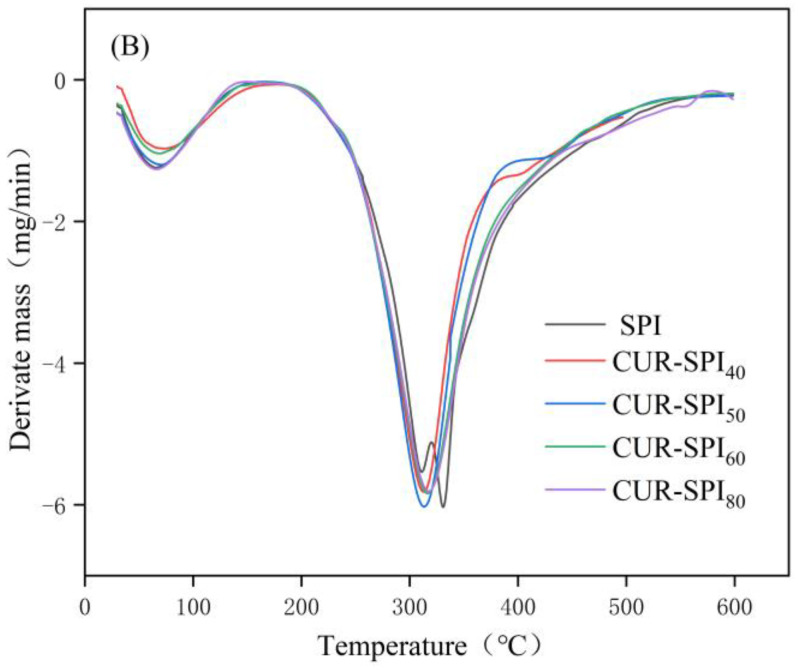
(**A**) TGA profiles and (**B**) DTG profiles of SPI and CUR-SPI gels.

Thermogravimetric analysis (TGA) and differential thermogravimetric analysis (DTG) were used to assess the thermal stability of the SPI and CUR-SPI gels, and the results are presented in Figure 4. As illustrated in Figure 4A, the initial mass loss stage, occurring below 100 °C, was attributed primarily to the evaporation of water molecules [13]. This observation was further supported by the DTG curves (Figure 4B), where the initial mass loss was evident at approximately 60 °C, indicating that water evaporation predominantly took place at approximately 60 °C. Importantly, the mass loss rate during the first stage (30–100 °C) was notably lower for the CUR-SPI complex gels than for the pure SPI gel (Figure 4B). This suggested that the addition of CUR effectively retained water within the gel. The second stage of mass loss occurred between 200 °C and 300 °C and was mainly attributed to the disassociation of the CUR-SPI interaction and the decomposition of CUR molecules, as the gel samples containing CUR-40 had a higher mass loss (Figure 4A). The third degradation stage at 320 °C corresponded to the decomposition of SPI protein backbones and the start of the volatilization of protein fragments [31]. Similarly, as shown in Figure 4B, the pure SPI gel displayed two peaks at approximately 310 °C and 330 °C, corresponding to the degradation of the main peptide chain of SPI [32]. However, after the addition of CUR, the CUR-SPI mixed gels exhibited only one peak, and all of them were below 320 °C. This finding suggested that the incorporation of CUR altered the ordered structure of the SPI. The mass loss rates of the SPI and CUR-SPI gels, occurring between 320 °C and 600 °C, were 38.33%, 30.29%, 31.99%, 35.6%, and 35.7%, respectively. This indicates that the final solid residue in the CUR-SPI mixed gels was greater than that in the pure SPI gel, with CUR-SPI_40_ having the highest residue. Generally, a higher TGA solid residue rate indicates less thermal decomposition, indicating greater thermal stability. This observation aligns with the notion that composite hydrogels with denser structures tend to yield more sample residue and require more external energy for thermal denaturation, thus exhibiting greater thermal stability [33]. In conclusion, the results of TGA and DTG analyses demonstrated that the thermal stability of SPI gels can be effectively enhanced by the addition of CUR. These findings are consistent with the work of Li et al. [19], who also identified the presence of hydrogen bonds in the interaction between SPI and TRC or TRIC. The improved thermal stability of SPI gel will be conducive to expanding its application scope, improving its processing performance, and enhancing the quality of the final product.

### 3.6. FT-IR Analysis

**Figure 5:**
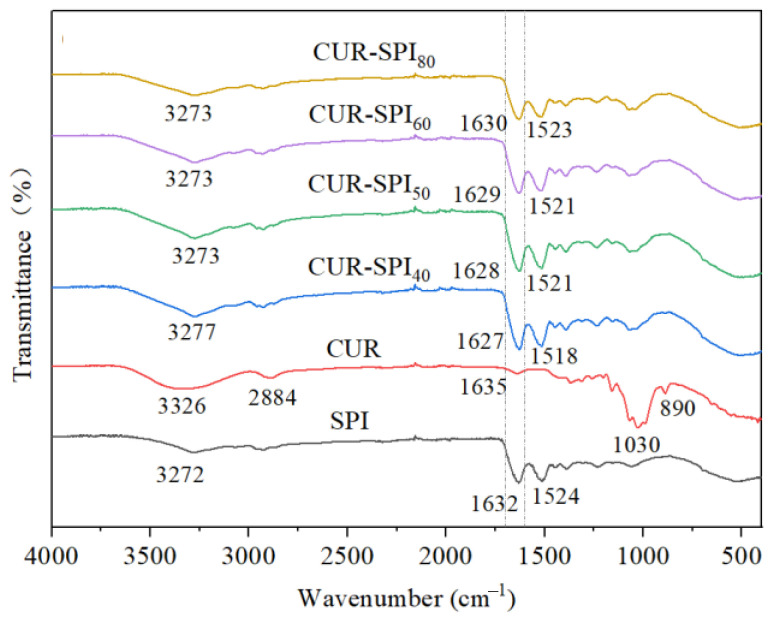
FT-IR spectra of SPI and CUR-SPI gels.

Fourier transform infrared (FT-IR) spectroscopy was employed in this study to assess the potential intermolecular interactions between SPI and CUR during gelation. As depicted in Figure 5, both the SPI and CUR-SPI gels exhibited similar IR spectra. Furthermore, compared with those of the pure SPI gel and CUR, no new absorption peaks were observed in the IR spectra of the CUR-SPI complex gels. This finding suggested that the interaction between SPI and CUR was primarily non-covalent in nature. In particular, Figure 5 clearly shows that the broad absorption band at approximately 3272 cm^−1^, attributed to the stretching vibrations of O-H and N-H groups in the pure SPI gel, shifted to 3273 or 3277 cm^−1^ in the CUR-SPI mixed gels. This shift indicates the presence of hydrogen bonds between SPI and CUR [34]. Similar observations have been reported in previous studies [13,14,15]. Furthermore, the band corresponding to the O-H and N-H bending vibrations in the CUR-SPI_40_ gel exhibited a larger red-shift or blue-shift, approximately 5 cm^−1^ more than the other CUR-SPI mixed gels did. This result demonstrated that CUR, which has an ordered triple-helix structure in bundles, formed stronger hydrogen bonding interactions with SPI than CUR, which has other helical conformations [19,25]. These findings align with the gel strength (Figure 1B), and TPA (Table 1) results. Additionally, amide bands I and II, appearing at approximately 1632 and 1524 cm^−1^, respectively, in the pure SPI gel [9], exhibited slight shifts toward lower wavenumbers in the CUR-SPI mixed gels. This indicates that the secondary structure of the SPI molecule underwent changes upon the addition of CUR.

### 3.7. Changes in SPI Structure During Gel Formation

**Figure 6:**
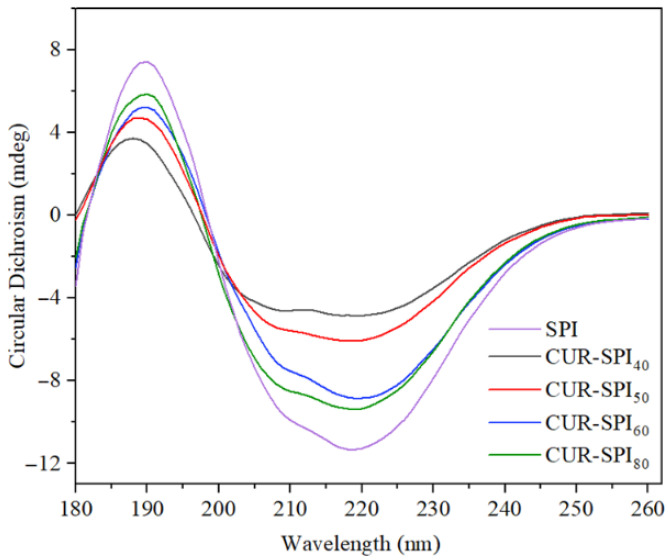
CD spectra of SPI and CUR-SPI gels.

CD spectroscopy is commonly employed to analyze the secondary structure of proteins [12]. Figure 6 illustrates the effects of CUR addition on the secondary structure of SPI. Both SPI gels, with and without CUR, exhibited two negative peaks at approximately 208 and 220 nm and a positive peak at approximately 192 nm, indicative of the α-helix structure in SPI [35]. However, compared to that of the pure SPI gel, the peak intensity of the α-helix structure in the CUR-SPI composite gels was notably lower. This reduction suggested that the incorporation of CUR led to a decrease in the α-helix content of the SPI molecules. A reduction in the α-helix content typically signifies a decrease in the ordered protein structure and an increase in protein unfolding, which is conducive to protein gelation [36]. Furthermore, Figure 6 clearly shows that the peak intensity of the α-helix structure in the CUR-SPI_40_ gel was lower than that in the other CUR-SPI composite gels. This finding emphasizes that, compared with CUR with other helical conformations, CUR obtained at 40 °C with bundles of triple helical structures was more effective at altering the secondary structure of SPI, resulting in a reduced α-helix content. These findings align with those of previous research by Jiang et al. [27].

**Figure 7:**
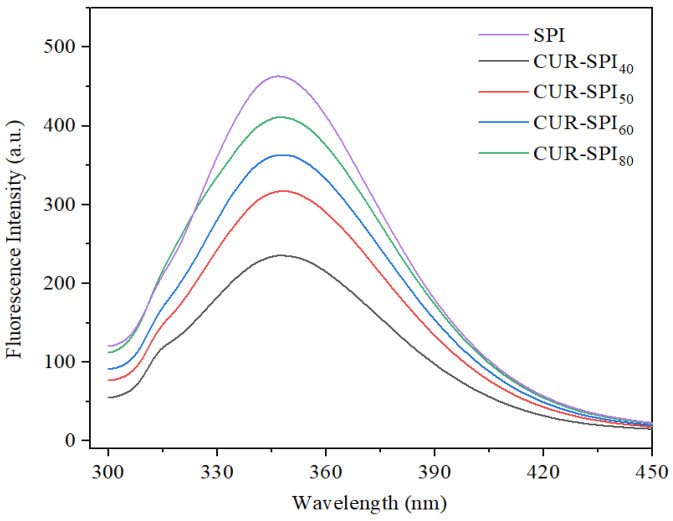
Intrinsic fluorescence spectra of SPI and CUR-SPI gels.

Tryptophan (Trp) is sensitive to polar microenvironments and is commonly used as an endogenous fluorescent probe to analyze changes in protein tertiary structure [12,37]. In this study, the effect of CUR with different helical conformations on the tertiary structure of SPI was examined using fluorescence spectroscopy, as shown in Figure 7. As expected, the addition of CUR, which has different helical conformations, obviously reduced the fluorescence intensity of Trp in the CUR-SPI composite gels to nearly 346.8 nm compared to that in the pure SPI gel. Additionally, the maximum emission wavelength (λ_max_) of the pure SPI gel exhibited a slight blue-shift after the incorporation of CUR. These observations suggest that CUR incorporation altered the conformation of SPI during the gelation process. Similar findings have been reported in previous studies [13,15,27]. Trp residues are typically located in hydrophobic environments within folded proteins, resulting in strong fluorescence. However, when proteins partially or fully unfold, Trp residues are exposed to hydrophilic environments, leading to a decrease in fluorescence intensity [12,38]. The data from this study indicate that the noncovalent interactions between SPI and CUR facilitated the unfolding of SPI molecules, exposing Trp residues to hydrophilic environments and thus reducing the fluorescence intensity. Notably, the fluorescence intensity of the CUR-SPI_40_ composite gel was considerably lower than that of the other CUR-SPI composite gels. This difference can be attributed to the stronger interactions between CUR obtained at 40 °C, which has bundles of triple helices, and SPI than between CUR and other helical conformations. In conclusion, the results shown in Figure 6 and Figure 7 demonstrate that the addition of CUR, particularly CUR obtained at 40 °C, with bundles of triple-helix structures, promotes the unfolding of SPI molecules, resulting in decreased α-helix content and exposure of Trp residues. This contributed to the formation of a gel network structure with enhanced gel strength (Figure 1B) and improved mechanical properties (Table 1).

## 4. Conclusions

In conclusion, this study demonstrated that the inclusion of CUR with various helical conformations can enhance the gelling properties of heat-induced SPI gels, including the WHC, gel strength, mechanical properties, viscoelasticity, and thermal stability. Furthermore, inclusion of CUR visibly improved the microstructures of heat-induced SPI gels. These improvements can be attributed to the enhanced hydrogen bonding interactions between CUR and SPI molecules. The addition of CUR induced changes in the secondary and tertiary structures of the SPI molecules, leading to the reduced α-helix content and the exposure of Trp residues. This, in turn, promoted the unfolding and cross-linking of SPI molecules. Notably, compared with those of other CUR-SPI composite gels, the CUR-SPI_40_ gel, which was formed by incorporating CUR with bundles of triple-helical structures into SPI, exhibited superior gel properties. It displayed a more compact and homogeneous three-dimensional gel network structure. Therefore, this study provides valuable insights for the development and design of new functional foods based on heat-induced SPI mixed gels, and the method’s cost-controllable raw materials. However, this study is not able to accurately regulate the conformation of CUR, which is also our next research direction.

## Figures and Tables

**Table 1:** Effects of CUR with different helical conformations on texture profiles of SPI and CUR-SPI gels (9% SPI).

Samples	Hardness (N)	Chewiness (N)	Springiness (%)	Cohesiveness
SPI	0.98 ± 0.02 ^d^	0.84 ± 0.02 ^d^	83.01 ± 2.25 ^cd^	0.905 ± 0.004 ^a^
CUR-SPI_40_	3.20 ± 0.10 ^a^	7.10 ± 0.01 ^a^	97.52 ± 2.26 ^a^	0.93 ± 0.024 ^a^
CUR-SPI_50_	1.61 ± 0.07 ^b^	2.25 ± 0.04 ^b^	91.62 ± 0.47 ^b^	0.906 ± 0.002 ^a^
CUR-SPI_60_	1.28 ± 0.07 ^c^	1.08 ± 0.01 ^c^	88.10 ± 2.54 ^bc^	0.889 ± 0.021 ^a^
CUR-SPI_80_	1.09 ± 0.02 ^d^	0.85 ± 0.01 ^d^	86.50 ± 0.72 ^cd^	0.895 ± 0.09 ^a^

Note: Each value was displayed as mean ± SD (*n* = 3), and different lowercase letters in the same column indicate significant differences (*p* < 0.05). The CUR-SPI_40_, CUR-SPI_50_, CUR-SPI_60_, and CUR-SPI_80_ were the CUR-SPI composite gels obtained at different temperatures (40 °C, 50 °C, 60 °C, and 80 °C).

## Data Availability

The original contributions presented in the study are included in the article, further inquiries can be directed to the corresponding authors.
